# En route to controlled catalytic CVD synthesis of densely packed and vertically aligned nitrogen-doped carbon nanotube arrays

**DOI:** 10.3762/bjnano.5.24

**Published:** 2014-03-03

**Authors:** Slawomir Boncel, Sebastian W Pattinson, Valérie Geiser, Milo S P Shaffer, Krzysztof K K Koziol

**Affiliations:** 1 Department of Organic Chemistry, Biochemistry and Biotechnology, Silesian University of Technology, Krzywoustego 4, 44-100 Gliwice, Poland; 2 Department of Materials Science and Metallurgy, University of Cambridge, 27 Charles Babbage Road, Cambridge CB3 0FS, United Kingdom; 3 Imperial College London, Department of Chemistry, London SW7 2AZ, United Kingdom

**Keywords:** carbon nanotubes, catalytic chemical vapour deposition, crystallinity, nitrogen doping, vertically aligned nanotube arrays

## Abstract

The catalytic chemical vapour deposition (c-CVD) technique was applied in the synthesis of vertically aligned arrays of nitrogen-doped carbon nanotubes (N-CNTs). A mixture of toluene (main carbon source), pyrazine (1,4-diazine, nitrogen source) and ferrocene (catalyst precursor) was used as the injection feedstock. To optimize conditions for growing the most dense and aligned N-CNT arrays, we investigated the influence of key parameters, i.e., growth temperature (660, 760 and 860 °C), composition of the feedstock and time of growth, on morphology and properties of N-CNTs. The presence of nitrogen species in the hot zone of the quartz reactor decreased the growth rate of N-CNTs down to about one twentieth compared to the growth rate of multi-wall CNTs (MWCNTs). As revealed by electron microscopy studies (SEM, TEM), the individual N-CNTs (half as thick as MWCNTs) grown under the optimal conditions were characterized by a superior straightness of the outer walls, which translated into a high alignment of dense nanotube arrays, i.e., 5 × 10^8^ nanotubes per mm^2^ (100 times more than for MWCNTs grown in the absence of nitrogen precursor). In turn, the internal crystallographic order of the N-CNTs was found to be of a ‘bamboo’-like or ‘membrane’-like (multi-compartmental structure) morphology. The nitrogen content in the nanotube products, which ranged from 0.0 to 3.0 wt %, was controlled through the concentration of pyrazine in the feedstock. Moreover, as revealed by Raman/FT-IR spectroscopy, the incorporation of nitrogen atoms into the nanotube walls was found to be proportional to the number of deviations from the sp^2^-hybridisation of graphene C-atoms. As studied by XRD, the temperature and the [pyrazine]/[ferrocene] ratio in the feedstock affected the composition of the catalyst particles, and hence changed the growth mechanism of individual N-CNTs into a ‘mixed base-and-tip’ (primarily of the base-type) type as compared to the purely ‘base’-type for undoped MWCNTs.

## Introduction

The doping of carbon nanotubes (CNTs) with boron [1–2], nitrogen [3–4] or phosphorus [5] atoms has been frequently used to enhance or tune their physicochemical properties. Among the elemental dopants, nitrogen emerges as of particular interest in electronics since N-CNTs should be characterized by a higher electrical conductivity (n-doping). Consequently, the significance of N-CNTs in a variety of electrical engineering applications has been continuously growing [6–7]. The enhancement of other properties of N-CNTs, including the chemical reactivity [8], the dispersibility in a variety of solvents/matrices [9], the structural strength [10] or the thermal conductivity [11] has been also reported. The field emission characteristics of N-CNTs have also been demonstrated to be superior to that of pristine CNTs [12]. Moreover, N-CNTs emerged as material of an improved capability to anchor metal nanoparticles through nitrogen coupling [13–14] and to form catalytically active centres, e.g., for the reduction of nitrogen oxides (N*_x_*O*_y_*) emission in exhausts [15]. A high degree of vertical alignment in the nanotube films (also called ‘carpets’ or ‘forests’) is a key aspect in numerous applications that gain from anisotropy, i.e., supercapacitors [16], counter electrodes [17], structural composites of enhanced thermal and electrical conductivity [18–19], superhydrophobic surfaces [20], separation membranes [21] and sensors [22]. As for aligned N-CNT arrays, to mention the most recent and prominent applications, they have shown to be suitable catalysts for the reduction of oxygen in alkaline fuel cells [23] or paracetamol sensors [24].

Nitrogen atoms can be incorporated into the CNT lattice trough either in situ or post-treatment strategies [25]. The former techniques are dominant and comprise primarily catalytic chemical vapour deposition (c-CVD) and its variations, which include bias or plasma enhancements, with typical parameters of the synthesis being the selection of the nitrogen source and/or the catalyst, and temperature. The literature survey (Table 1) shows that the N-doping of CNTs usually induced lattice deformations, i.e., the formation of regular and irregular compartments that replace or accompany the multi-wall structure. Those defects, e.g., ‘bamboo’-and ‘nanobell’-morphologies could translate further into twists and corrugations, and as a consequence, into a lesser degree of alignment of the arrays. Additionally, the incorporation of nitrogen atoms was found to be the driving force in the formation of defect sites in the carbon sp^2^-network [26].

**Table 1:** Literature overview of representative synthetic pathways toward N-CNTs.

method	C/N feedstock	catalyst (precursor)	*T*, °C	product	N content, wt %	ref.

arc discharge	graphite, melamine	Ni–Y	—	N-SWCNT	1.0	[27]
arc discharge	graphite, N_2_	Fe–Ni–Co	—	N-CNT	4.6–13.7	[28]
arc discharge	graphite, N_2_	Fe_2_O_3_, Co_2_O_3_, NiO	—	N-CNT	unknown	[29]

high-pressure CO conversion (HiPCO)	graphite, N_2_	-	1300	N-CNT, nanofibers	3.0–13.0	[30]

c-CVD	CH_3_CN	Co–Mo/MgO	700	bamboo-like N-CNT	12.0	[31]
c-CVD	CH_3_CN CH_3_CN + H_2_	Fe–MgO	850	bamboo-like N-CNT bamboo-like N-CNT	0.94 2.62	[32]
c-CVD	ethylenediamine	Co, FeCp_2_	780 860 980 1080	irregular N-CNT pearl-like nanobells bamboo-like N-CNT bamboo-like N-CNT	24.45 22.42 19.56 18.77	[33]
c-CVD	NH_3_	iron(II) phthalate, SiO_2_	850	aligned bamboo-like N-CNT	9.0	[34]
c-CVD	C_2_H_2_, NH_3_	Fe(CO)_5_	750 850 950	bamboo-like N-CNT	2.8 4.2 6.6	[35]
c-CVD	CH_3_CN	Co–Ni/SiO_2_	800	twisted bundles N-MWNT	3.0	[36]
c-CVD	THF, CH_3_CN	Fe(acac)_3_	950	bamboo-like N-MWNT	16.0–20.0	[37]
c-CVD	1. propylene, 2. CH_3_CN 1. CH_3_CN, 2. propylene	Al_2_O_3_	800	aligned N-CNT	3.2 3.5	[38]
c-CVD	C_2_H_2_, NH_3_	Fe/SiO_2_	850	bamboo-like N-CNT	0.4–2.4	[39]
c-CVD	CH_4_, NH_3_ C_2_H_2_, NH_3_	Fe/Si	900 1000 1100	bamboo-like N-CNT	2.0–6.0	[40]
c-CVD	CH_3_CN	Al_2_O_3_	800	aligned N-CNTs within Al_2_O_3_ pores	unknown	[41]
c-CVD	pyridine pyrimidine	FeCp_2_	600–900	aligned bamboo-like N-CNT	1.0–2.0 3.2	[42]
c-CVD	CH_4_, NH_3_, N_2_	Ni–Fe	720–810	bamboo-like CNT with graphene sheets	0–4	[43]

bias CVD	CH_4_, N_2_	Fe–Ni	500	bamboo-like N-CNT	≈15	[44]

plasma CVD	CH_4_, NH_3_	SiO_2_/Si	450	N-SWCNT	0–4	[45]
plasma CVD	graphite, N_2_	Ni	650	aligned bamboo-like N-CNT	20.0–30.0	[46]

magnetron sputtering	SWNTs, N_2_^+^	—	—	→ N-SWCNT → amorphous	450 ppm	[25]

low-energy ion irradiation	MWNTs, N_2_^+^	—	—	N-MWCNTs	0.58	[47]

We have previously reported that control over *chirality* of individual nanotube walls can be achieved by the selection of the nitrogen source, which is related to the stability of Fe_3_C nanoparticles as nucleation sites of the nanotubes growth in c-CVD process [48–51]. Here, we track how the parameters of a c-CVD synthesis, which employs pyrazine (Pz) as the precursor of nitrogen-based compounds, affect growth and properties of N-CNTs on the macro-, nano- and atomic scales. The growth kinetics, the morphology, and the areal density, and physical/spectral properties of the N-CNT products were studied as a function of the growth time, the temperature and the composition of the supply injected to the quartz-tube reactor.

## Results and Discussion

**Figure 1:**
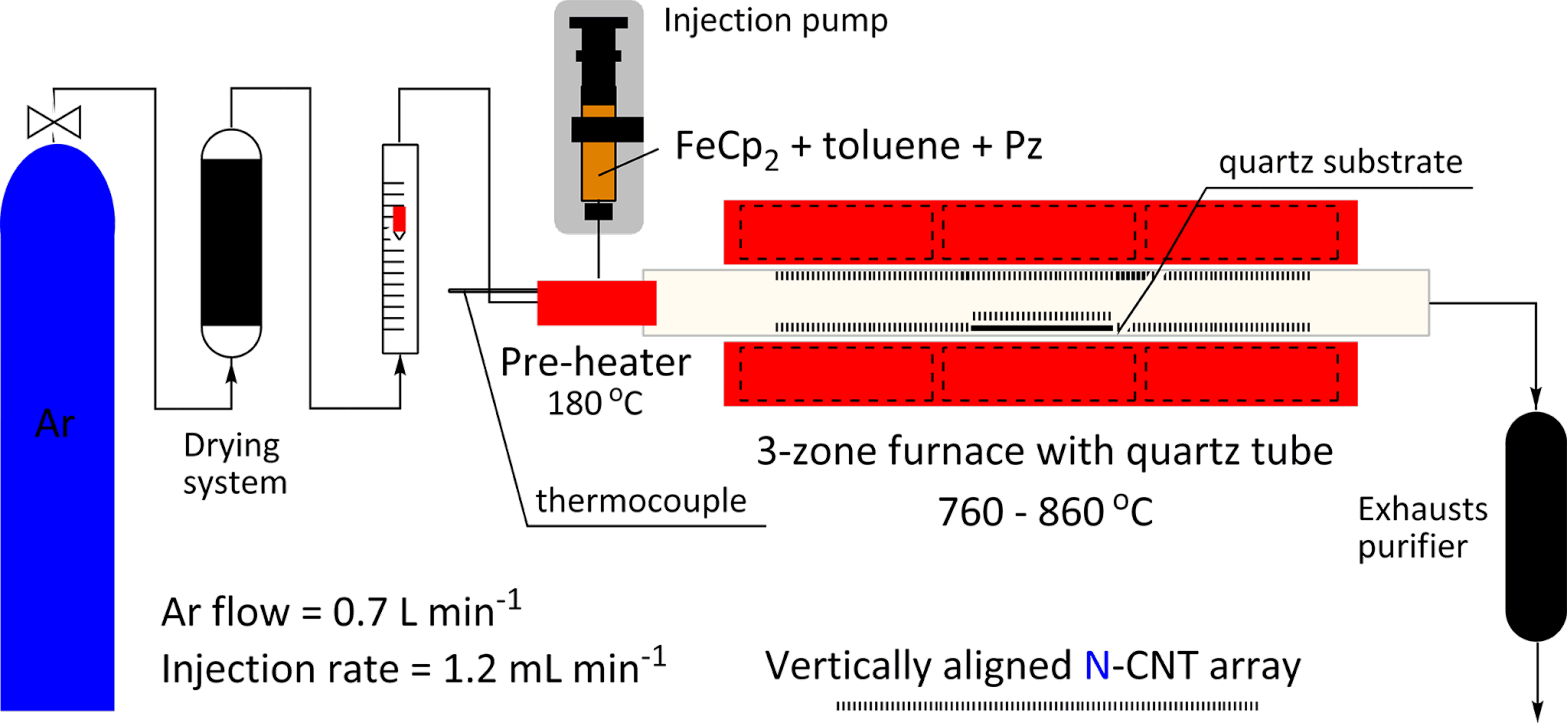
Injection c-CVD furnace and constant parameters for the synthesis of aligned N-CNT arrays.

**Synthesis of N-CNTs (and MWCNTs).** The synthesis of different types of N-CNT was carried out by using a horizontal injection c-CVD furnace (Figure 1, see ‘Experimental’ for full experimental data).

**Scheme 1:**
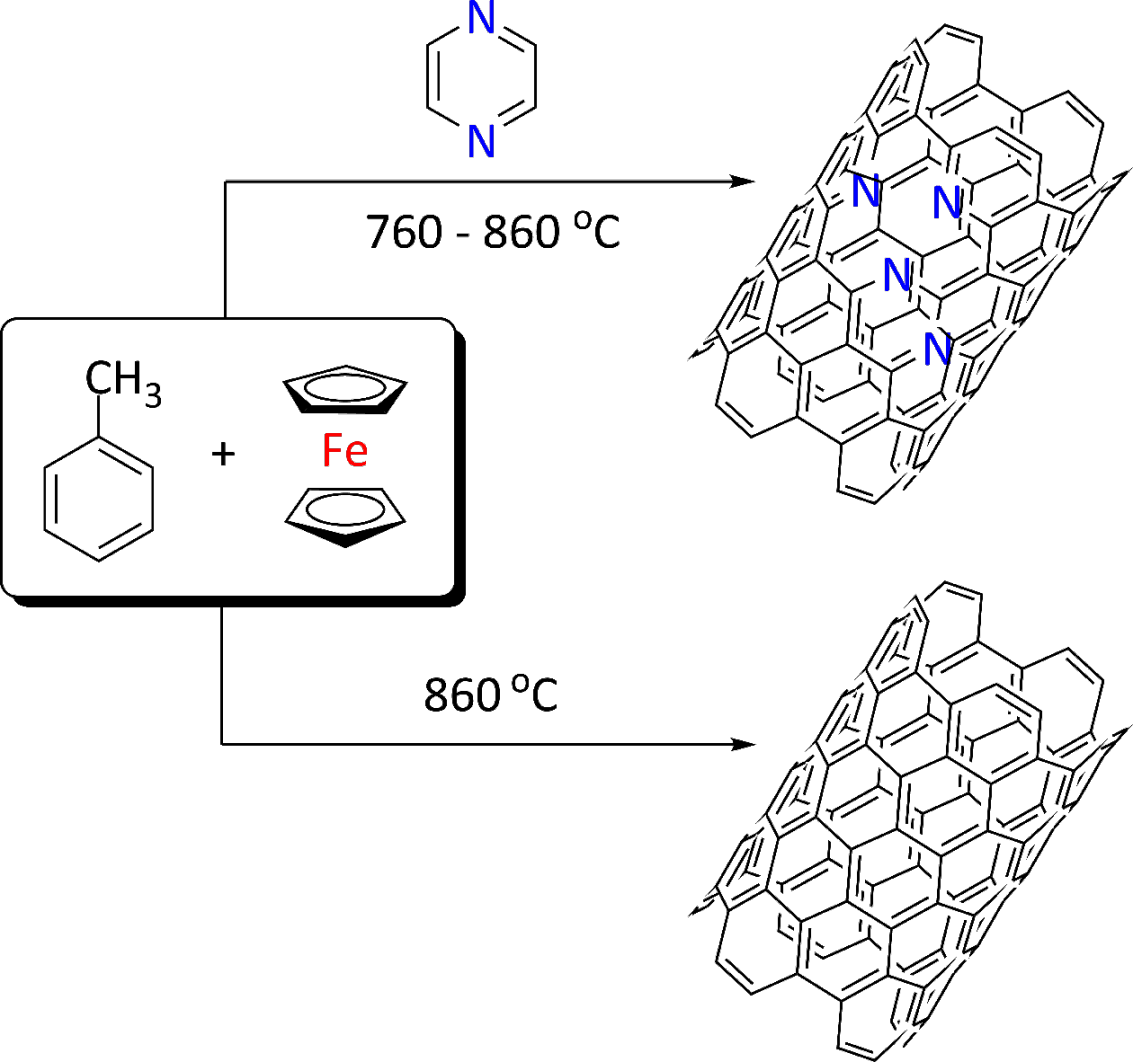
Schematic reactions in the synthesis of N-CNTs and MWCNTs; for clarity only the outer nanotube walls are shown.

The conditions of the synthesis were analogous to those applied for the catalytic growth of pristine MWCNT arrays (which were also synthesized here for comparison and referred to as *Ref. Synthesis*, Scheme 1), except that Pz, – the additional heteroaromatic nitrogen precursor, which decomposes into acetylene, hydrogen cyanide and cyanoacetylene [52–56] at 760 °C – was introduced into the feedstock. Pz was an immediate selection since it is well-soluble (85 g/100 g at room temperature) in toluene (PhMe, the primary carbon source), and has a boiling point (bp) of 115 °C, which is just 5 °C above the bp of PhMe. N-CNTs (and MWCNTs) grew both on the quartz substrates inserted into the centre of the furnace as well as on the internal wall of the quartz reactor.

**Optimising the conditions.** In order to establish conditions for a high growth rate of N-CNTs and the highest achievable alignment as well as a high density of the N-CNTs arrays, temperature and composition of the feedstock were scanned as variable parameters (Table 2). The parameters used were set progressively according to the emerging results. The starting point in the optimisation (*Synthesis I*) was based on the most suitable conditions for the synthesis of MWCNT carpets, i.e., 860 °C and [FeCp_2_] = 2 wt %. In case of the synthesis of N-CNTs, PhMe was partially replaced by Pz to 19.6 wt %. Then, temperature and [FeCp_2_] were kept constant, while [Pz] was gradually decreased (*Synthesis II*, *III*). Based on the SEM analysis (qualitative inspection of the alignment and purity of the product), parameters from *Synthesis II* were selected as the most appropriate for the further synthesis of aligned N-CNT arrays free from carbonaceous particles. Therefore next, temperature and [Pz] were left unchanged, while [FeCp_2_] was increased to 9.6 wt % (*Synthesis IV*). At those settings, the synthesis yielded clean and thinner nanotubes, but not dense arrays. Hence at a [Pz] value of 5 wt % and a [FeCp_2_] value of 9.6 wt %, the temperature was lowered to 760 °C (*Synthesis V*). Here, the nanotubes were visually purer as compared to the previous batch and the density of the N-CNT arrays was very high. Eventually, the temperature was set to remain unchanged, whereas [FeCp_2_] was decreased again to 2 wt % (*Synthesis VI*). Under these conditions, the obtained nanotube arrays were characterized by a higher alignment and a high areal density. Further, [Pz] was increased gradually (*Synthesis VII, VIII*), which led to the compromise between a high alignment and a high areal density. In a control experiment at 660 °C, in which [FeCp_2_] was also increased (*Synthesis IX*), no nanotubes could be identified as the product.

**Table 2:** Composition of the feedstock and growth temperatures in the syntheses of N-CNTs. The growth time was equally 4 h in all of the syntheses.

no.	*T*, °C	[PhMe], wt %	[Pz], wt %	[FeCp_2_], wt %

I	860	78.4	19.6	2.0
II	860	93.2	4.8	2.0
III	860	97.0	1.0	2.0
IV	860	85.2	5.2	9.6
V	760	85.4	5.0	9.6
VI	760	93.0	5.0	2.0
VII	760	68.0	30.0	2.0
VIII	760	53.0	45.0	2.0
IX	660	56.0	40.0	4.0
Ref.	860	95.4	—	5.6

**Macroscopic properties and elemental composition.** The observation of the N-CNTs at the macroscopic level revealed them as a harder and more brittle material as MWCNTs, which suggested either alterations in the structure of the catalyst nanoparticles and/or of the nanotube themselves. Nevidomskyy et al. reported in their ab-initio studies that nitrogen incorporation into SWCNTs could cause the formation of covalent junctions between neighbouring tubes if the nitrogen atoms were opposed to each other [57]. Therefore, if the density of inter-tube bonds is sufficiently high, a tightly-packed bundle of covalently cross-linked nanotubes (analogous to the cross-linking of polymer chains) with substantially changed mechanical properties, compared to MWCNT arrays, could be synthesized. The aligned MWCNT arrays were soft and could be easily peeled off from the quartz reactor in large pieces by using a razor blade, whereas the N-CNTs were peeled off as powder. Also, a drastic increase in the adhesion of N-CNT arrays to the quartz substrates as compared to pristine MWCNT films was noticeable because of changes in the structure and composition of the catalyst nanoparticles.

The contents of C, H, N and Fe, and empirical formulae (calculated per 1000 atoms) that were derived from the elemental analysis of the CNTs, as well as the respective feedstock compositions are presented in Table 3. The Fe content was determined from the amount of Fe_2_O_3_, which was found to be the sole residual material after the combustion in air after completion of the TG analysis. The iron content in the N-CNT product was higher than in the MWCNTs, apart from *Syntheses I*–*III*. The overall conversion of the initial carbon (in all carbon-bearing starting reactants) into carbon in nanotubes was different for MWCNTs and N-CNTs and was found to depend upon: 1) [FeCp_2_] as a crucial catalyst for the growth of nanotubes, and 2) [Pz] as the source of nitrogen compounds that stabilize the iron carbide phase. For these two extreme cases, the yield of nanotubes per amount of carbon was 8.9 and 36.6% for N-CNTs (*Synthesis VII*, [Pz] = 30%, [FeCp_2_] = 2%, [PhMe] = 68%) and MWCNTs, respectively. The other yields lie in this range, e.g., for *Synthesis V* ([Pz] = 5%, [FeCp_2_] = 9.6%, [PhMe] = 85.4%) the yield per amount of carbon was 27.1%.

**Table 3:** Elemental composition of the feedstock vs N-CNT products from *Syntheses I-VIII* in a comparison with MWCNTs.

no.	*T*, °C	feedstock, wt %				N-CNT product, wt %				formula
		C	H	N	Fe	C	H	N	Fe	

I	860	84.6	7.9	6.9	0.6	97.8	0.4	0.8	1.0	C_947_H_46_N_7_
II	860	89.3	8.4	1.7	0.6	97.0	0.4	0.2	2.4	C_951_H_47_N_2_
III	860	90.5	8.6	0.3	0.6	97.9	0.4	0.0	1.7	C_953_H_47_
IV	860	87.1	8.2	1.8	2.9	91.3	0.3	0.0	8.4	C_962_H_38_
V	760	87.1	8.2	1.8	2.9	84.1	0.3	0.4	15.2	C_955_H_41_N_4_
VI	760	89.2	8.4	1.8	0.6	92.0	0.3	0.6	7.1	C_957_H_37_N_6_
VII	760	81.4	7.5	10.5	0.6	91.5	0.3	1.4	6.8	C_950_H_37_N_13_
VIII	760	76.7	7.0	15.7	0.6	91.1	0.3	3.0	5.6	C_937_H_37_N_26_
Ref.	860	90.8	8.6	0	0.6	96.5	0.3	0	3.2	C_964_H_36_

**Figure 2:**
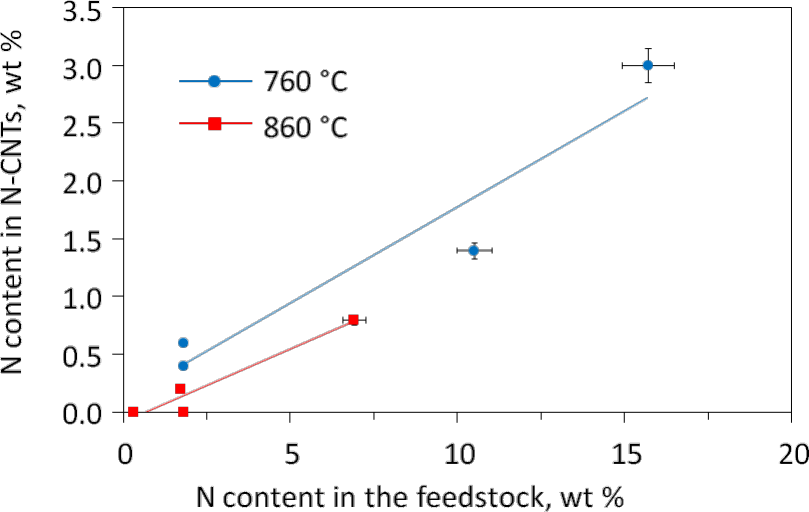
Relationship between the nitrogen content in the N-CNTs products and in the feedstock.

The nitrogen content of the N-CNTs was found to correlate with that in the feedstock, both at 760 °C and 860 °C (Figure 2). Note that the higher incorporation of nitrogen into the nanotube lattice occurred at the lower temperature. Also noteworthy is a similar hydrogen content in all N-CNTs and MWCNT. All nanotube products contained 0.3–0.4 wt % H, which is assumed to be in a form of nanotube H-terminations and other possible C–H functions localized at structural defects.

**Figure 3:**
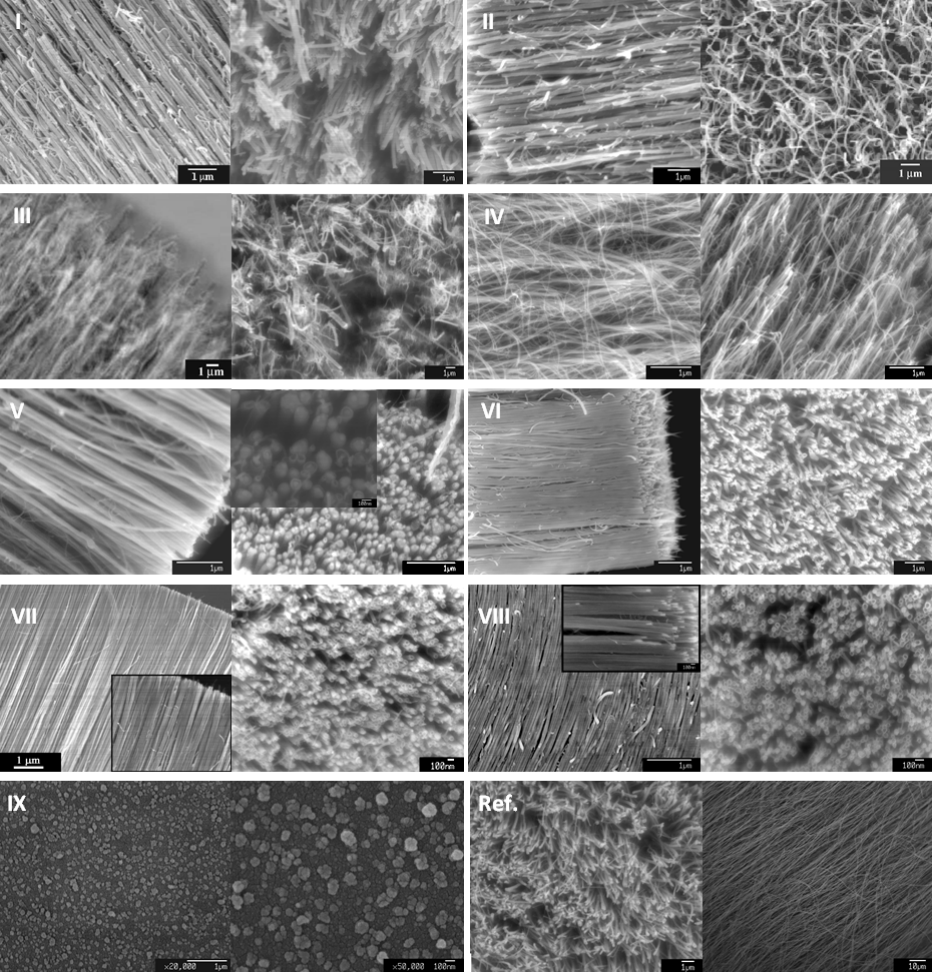
SEM examination of the N-CNTs from *Syntheses I–IX* and MWCNT from *Ref. Synthesis*.

**Morphology of N-CNTs.** Raw products from each of the N-CNTs syntheses were examined with respect to the degree of alignment and areal density of the nanotubes, the purity (presence/absence of carbonaceous particles), the structure of the individual nanotubes (with a special emphasis on their straightness), and the quantitative analysis of the outer and inner diameters (OD, ID) of the nanotubes. Although the raw products were analysed, practically no amorphous carbon was observed for all of the N-CNT arrays of high quality. SEM images of the products from *Syntheses I–IX* are shown in Figure 3. In general, N-CNT films of different thicknesses were obtained depending on the concentration of the nitrogen species in the hot zone, which act as growth inhibitors. *Synthesis I* and *III* furnished nanotubes of small diameters that were twisted around the thicker ones. A lower degree of alignment, with abundant entangled nanotubes at the surface of the films in the products from *Synthesis I–IV* was evident. In the N-CNTs from *Synthesis V* the degree of alignment was higher, but some spiral and entangled nanotubes were also present (see Figure S1, Supporting Information File 1). In this case the nanotubes were nevertheless densely packed and of a narrow range of diameters. Eventually, it was confirmed that *Synthesis V* yielded the most dense N-CNT arrays of 5 × 10^8^ nanotubes per mm^2^ while the highest value of areal density of MWCNTs reported up-to-date was 4.9 × 10^8^ nanotubes per mm^2^ [58]. However, this value was found for the hydrogen-assisted growth from ethylene with an Fe/Al_2_O_3_ catalyst that was pre-treated with hydrogen. This synthesis yielded nanotubes whose thickness was about half to a third the thickness of the CNTs synthesized here. Nonetheless, it must be emphasized that the value of 5 × 10^8^ nanotubes per mm^2^ that cover 27% of the accessible area is still below the theoretically achievable areal density of the nanotubes (91%). In turn, SWCNT arrays as less geometrically restricted ‘forests’ can be grown up to 1.64 × 10^11^ nanotubes per mm^2^ [59]. Moreover, for the N-CNTs grown in *Synthesis V* the highest Fe content was found, and in the SEM imaging (in which pre-ultrasonication was not required) the catalyst nanoparticles can be still seen as ‘corks’ in the ‘elephant trunks’. An image of such a magnified bottom-view of the most dense N-CNT arrays from *Synthesis V* is an inset in Figure 3 in the corresponding window.

*Synthesis VI* resulted in well-aligned nanotubes with a narrow range of diameters and clearly visible open nanotube tips. *Synthesis VII* and *VIII* yielded densely packed (up to 5 × 10^7^ nanotubes per mm^2^), well-developed nanotubes with the highest degree of alignment. For comparison, for MWCNT arrays of ca. 5 × 10^6^ nanotubes per mm^2^ were found, and the misalignment was significantly higher. As mentioned earlier, when the temperature of the growth process was reduced (*Synthesis IX*), N-CNTs could not be detected and only catalyst particles of different heights and diameters were found as bright spots (‘seeds’) on the quartz substrate. In conclusion, the highest ‘quality’ of the nanotube arrays, i.e., no waviness, and a high density of nanotubes was found in the products from *Syntheses VII* and *VIII*. One hundred different nanotubes that were randomly selected from the TEM micrographs were measured. Different growth temperatures and concentrations of the feedstock ingredients influenced the average outer diameters (OD_av_) of N-CNTs, which ranged from 26 to 161 nm. The summary of the OD measurements is compiled in Table 4.

**Table 4:** Summary of the results from measurements of OD N-CNTs’ from *Syntheses I–VIII* and MWCNTs from *Ref. Synthesis*.

*Synthesis*	*I*	*II*	*III*	*IV*	*V*	*VI*	*VII*	*VIII*	*Ref.*

*T*, °C	860	860	860	860	760	760	760	760	860
[Pz], wt %	19.6	4.8	1.0	5.2	5.0	5.0	30.0	45.0	0.0
[FeCp2], wt %	2.0	2.0	2.0	9.6	9.6	2.0	2.0	2.0	2.0

N content, wt %	0.8	0.2	0.0	0.0	0.4	0.6	1.4	3.0	0.0

min. OD, nm	20	10	13	10	5	7	5	12	45

max. OD, nm	236	224	461	61	64	93	95	99	127

OD_av_, nm	102 ± 48	84 ± 44	161 ± 105	34 ± 13	26 ± 15	43 ± 21	43 ± 22	49 ± 19	83 ± 19

**Figure 4:**
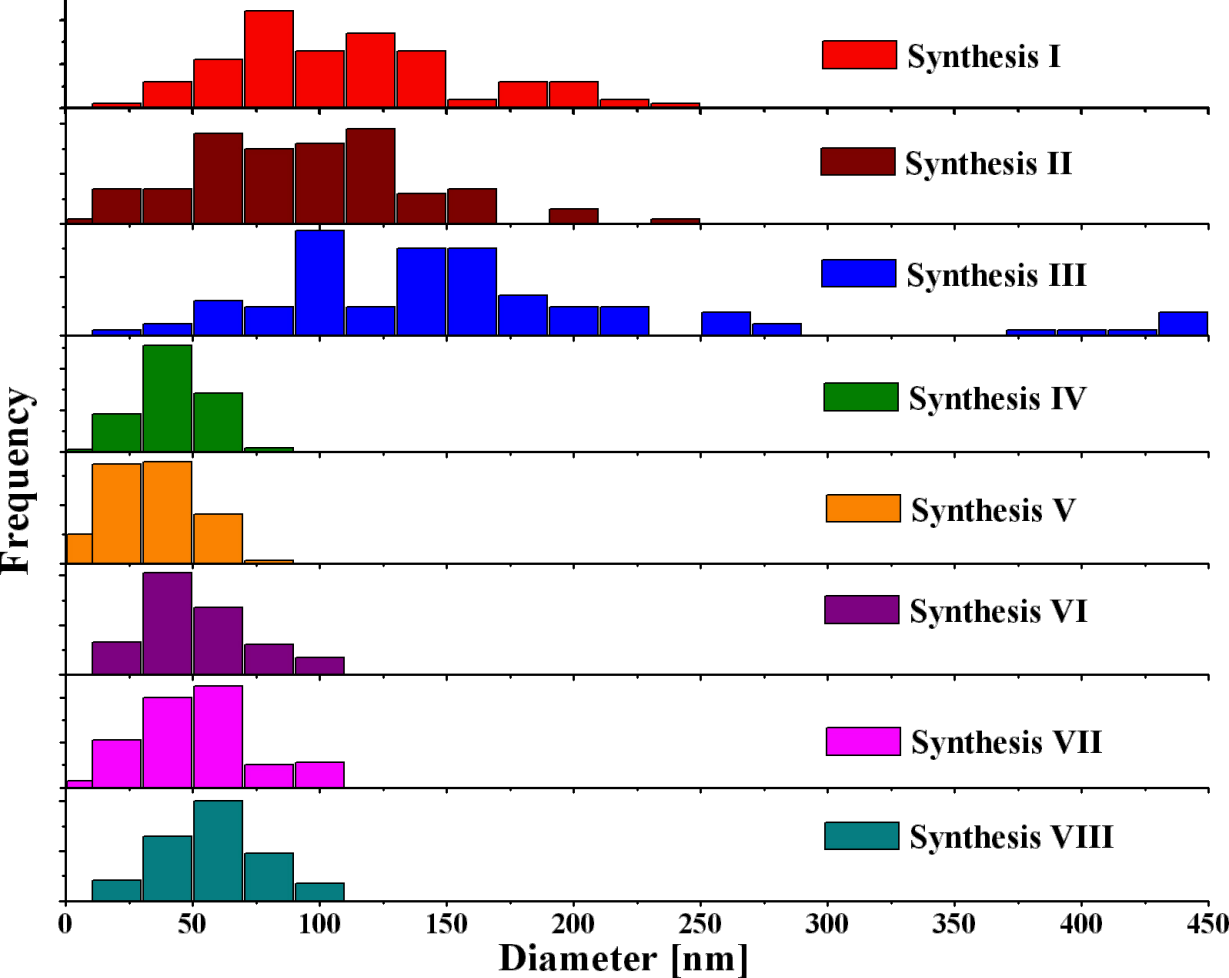
Histograms of the outer diameters of N-CNTs from *Syntheses I–VIII*.

At 860 °C, and constant and low [FeCp_2_], OD_av_ of N-CNTs was found to be between 84 and 161 nm. A significant decrease in OD_av_ was observed after increasing [FeCp_2_] while keeping the temperature of growth at 860 °C. In *Syntheses IV* and *V* one can observe that decreasing the temperature from 860 to 760 °C caused a drop of OD_av_ from 34 to 26 nm. Lowering [FeCp_2_] from 9.6 to 2.0 wt % at 760 °C caused an increase in OD_av_ from 26 to 43 nm. No obvious relationship between the concentration of nitrogen in the feedstock and the value of OD_av_ of the nanotubes was found. The medium values of OD_av_ for N-CNTs from *Syntheses VI–VIII* were around 45 nm, which is about half of those of pure MWCNTs (83 nm). The distribution of the outer diameters of the N-CNTs is presented in Figure 4. As can be seen, it was found to be broad at higher temperatures but it is narrowed down when increasing [FeCp_2_] in the feedstock and decreasing the temperature.

**Figure 5:**
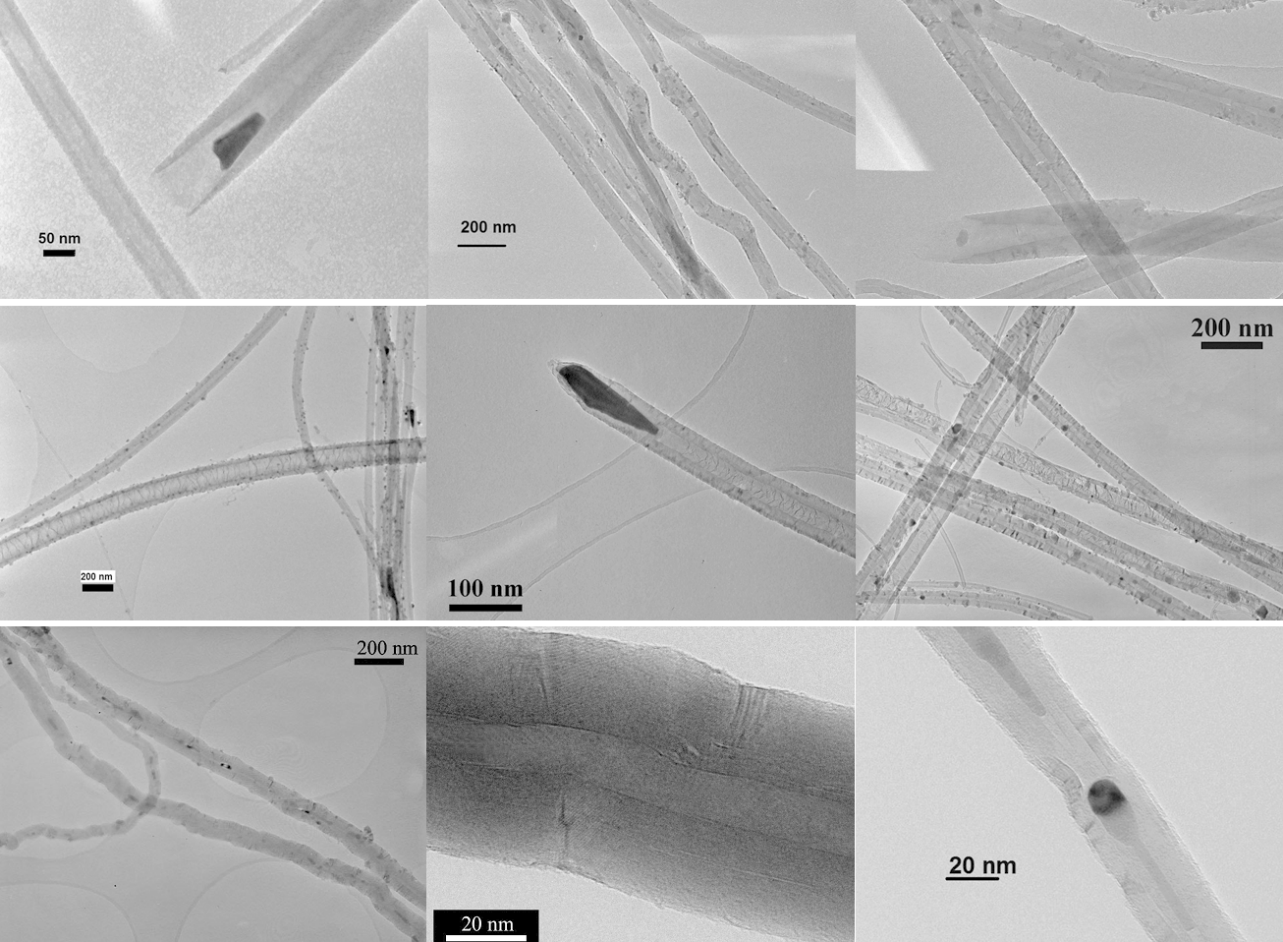
TEM images of: N-CNTs from *Synthesis VII* (**upper panel**) – straight N-CNTs; magnified views reveal ‘bamboo’-like (cone-like) periodic structures; N-CNTs from *Synthesis VIII* (**middle panel**) – straight N-CNTs of a compartmental (‘membrane’-like) morphology and bearing large catalyst nanoparticle residues at the nanotube ends and smaller ones on the outer walls, occasionally embedded in between the wall filaments; MWCNTs from *Ref. Synthesis* (**lower panel**) – nanotubes composed of irregular walls full of corrugations and kinks with a core discontinuously filled with metallic nanoparticles.

It has been reported that higher temperatures of the nanotube growth increased both the OD_av_ of the nanotubes as well as the range of the ODs [60–61]. In the case of undoped carbon nanotubes, increasing [FeCp_2_] resulted in an increase of the diameter of the MWCNTs [55]. This phenomenon is related to Ostwald ripening [62–64]. However, the opposite was found in N-CNTs, which could indicate a different mechanism of growth, which will be discussed further below. Also, the inner diameters of the nanotubes from *Syntheses VI–VIII* was measured based on TEM. The average value was 22 ± 5 nm, which is larger than that of pure MWCNTs (10 ± 4 nm). The increased ID of N-CNTs must be related to a larger size of the catalyst particles, which are located at the growth end of the nanotubes. Additionally, N-CNTs have fewer walls. Typically about 30 walls are present in the nitrogen tubes compared to around 100 in the pure MWCNTs. N-CNTs produced in *Syntheses VII* and *VIII* were found to be smoother (Figure 5) as compared to MWCNTs. A feature exclusive for N-CNTs is that their inner core is not continuously hollow as it is in MWCNTs, but is separated by discrete layers of distorted graphene across the core perpendicular to the tube axis.

**Figure 6:**
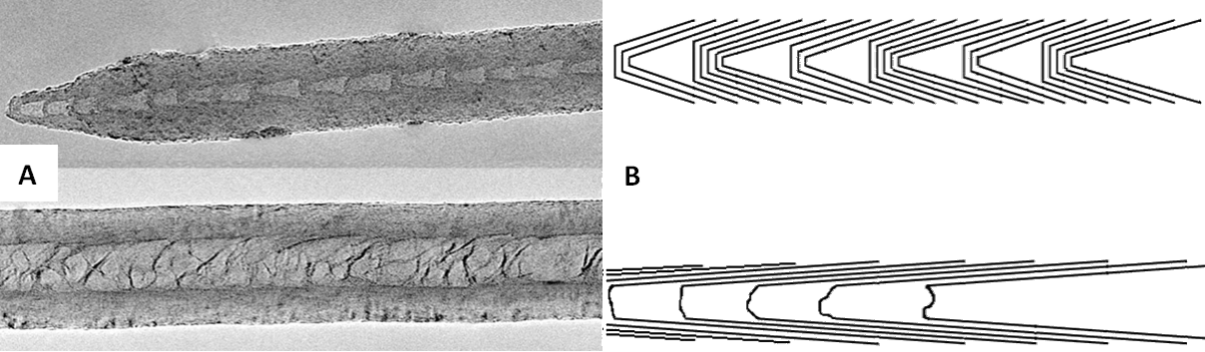
(**A**) TEM micrograph comparing two distinguishable types of nanotube morphologies: *top* – ‘bamboo’-like (*Synthesis VII*) and *bottom* – ‘membrane’-like (*Synthesis VIII*); and (**B**) graphical representations thereof.

No significant kinks in the nanotube walls could be observed in the TEM images of N-CNTs from *Synthesis VII* (Figure 5, upper panel). The outer walls were typically smooth (just a few wavy nanotubes among hundreds were found). A careful look at the magnified images reveals that the nanotube cores are characterized by a periodic internal morphology, i.e., ‘bamboo’-like (or cone-like) of a distinctive regularity. Unlike pristine MWCNTs, which have iron particles encapsulated inside the core, the channels of the N-CNTs were free from metal nanoparticles suggesting that, for instance, a partial removal of the catalyst residues could occur while removing N-CNTs from the quartz surface. Representative TEM images of N-CNTs from *Synthesis VIII* are shown in the middle panel of Figure 5. Numerous characteristic graphene layers, which divide the nanotube core into densely distributed compartments, could be observed here. The N-CNTs were grown as straight as in the case of nanotubes from *Synthesis VII*. In this case however, larger metal catalyst nanoparticle residues (dark spots) can be visible at the nanotube ends. It must be emphasized that Fe_3_C is a brittle material, which can be easily withdrawn from the nanotube tips during ultrasonication, and also possibly in other mechanical operations. Moreover, there are a few smaller particles on the outer surface of the nanotubes, which are not likely to take part in the nanotube growth. Also, catalyst nanoparticles were found to be embedded in between the nanotube wall filaments. The removal of the catalyst particles either during scraping off the nanotubes from the substrate surface or during the TEM sample preparation in the ultrasonic bath left a wedge shaped cavity at the nanotube end. This behavior has never been observed for MWCNTs. A summary of representative and dominating nanotube morphologies from *Synthesis VII* and *Synthesis VIII* with the corresponding models is presented in Figure 6.

**Figure 7:**
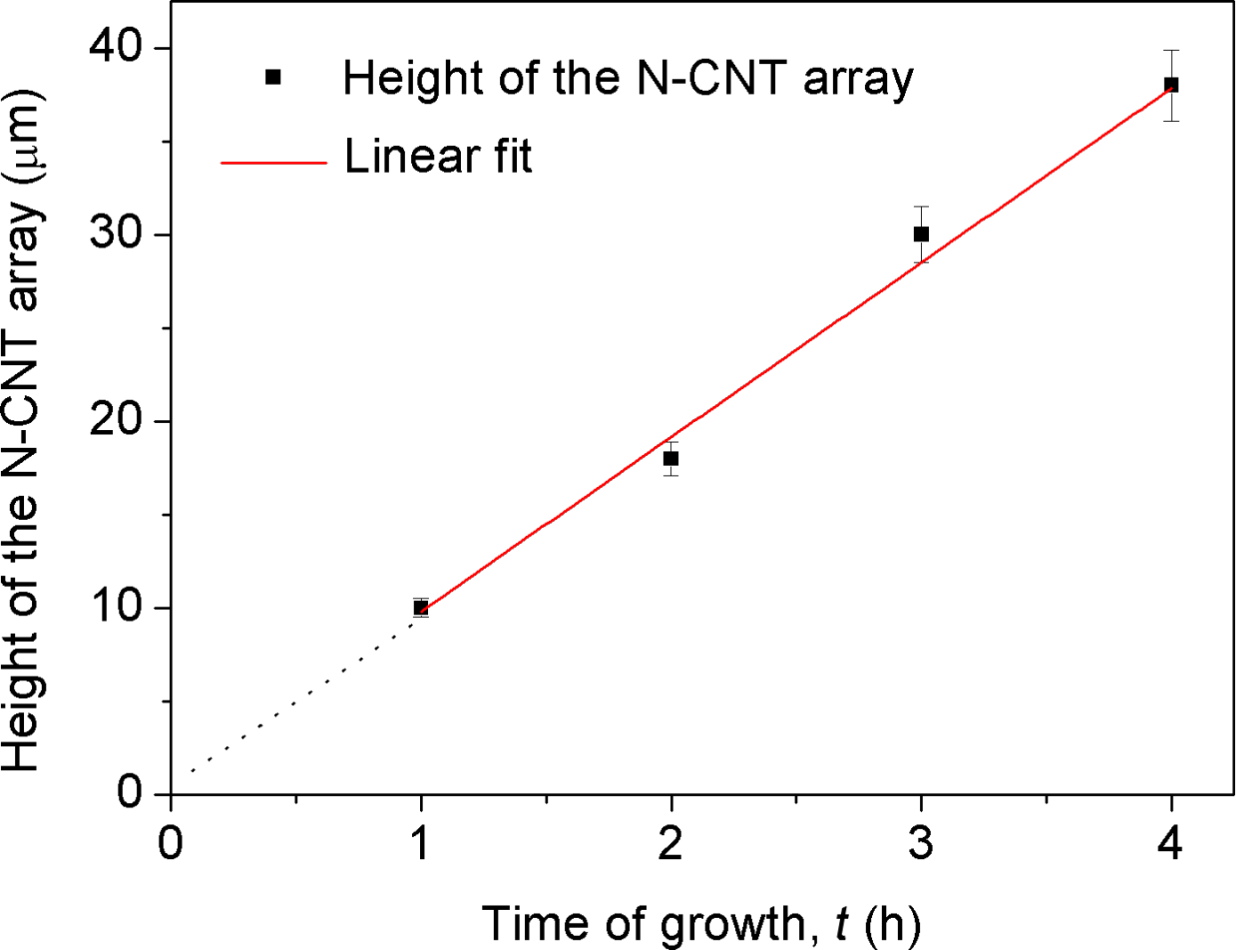
The height of the N-CNTs array vs time of growth.

**Growth rate of N-CNT arrays.** In order to analyse the growth rate of N-CNTs four runs were carried out with different times of synthesis, and with high [Pz] as the growth-retardant and the lowest concentration of catalyst precursor; namely 1, 2, 3 and 4 h at 760 °C, and at the following composition of the feedstock: [PhMe] = 68.0, [Pz] = 30.0, and [FeCp_2_] = 2.0 wt.%. In this kinetic experiment it was demonstrated that for the period of 4 h the growth rate of N-CNTs remained linear at approximately 10 ± 1 µm·h^−1^ (Figure 7).

The overall growth of nanotubes was substantially inhibited by the presence of nitrogen compounds in the growth zone, with the growth rate for pristine MWCNTs being about 20 times higher under otherwise equivalent conditions. Obviously, the growth rates of other N-CNT arrays lie in the range depicted above, which confirms [Pz] and [FeCp_2_] to be the critical parameters for the growth rate of nanotube arrays. The linear fit function passes through the point of origin, which suggests a short time of nucleation.

**Figure 8:**
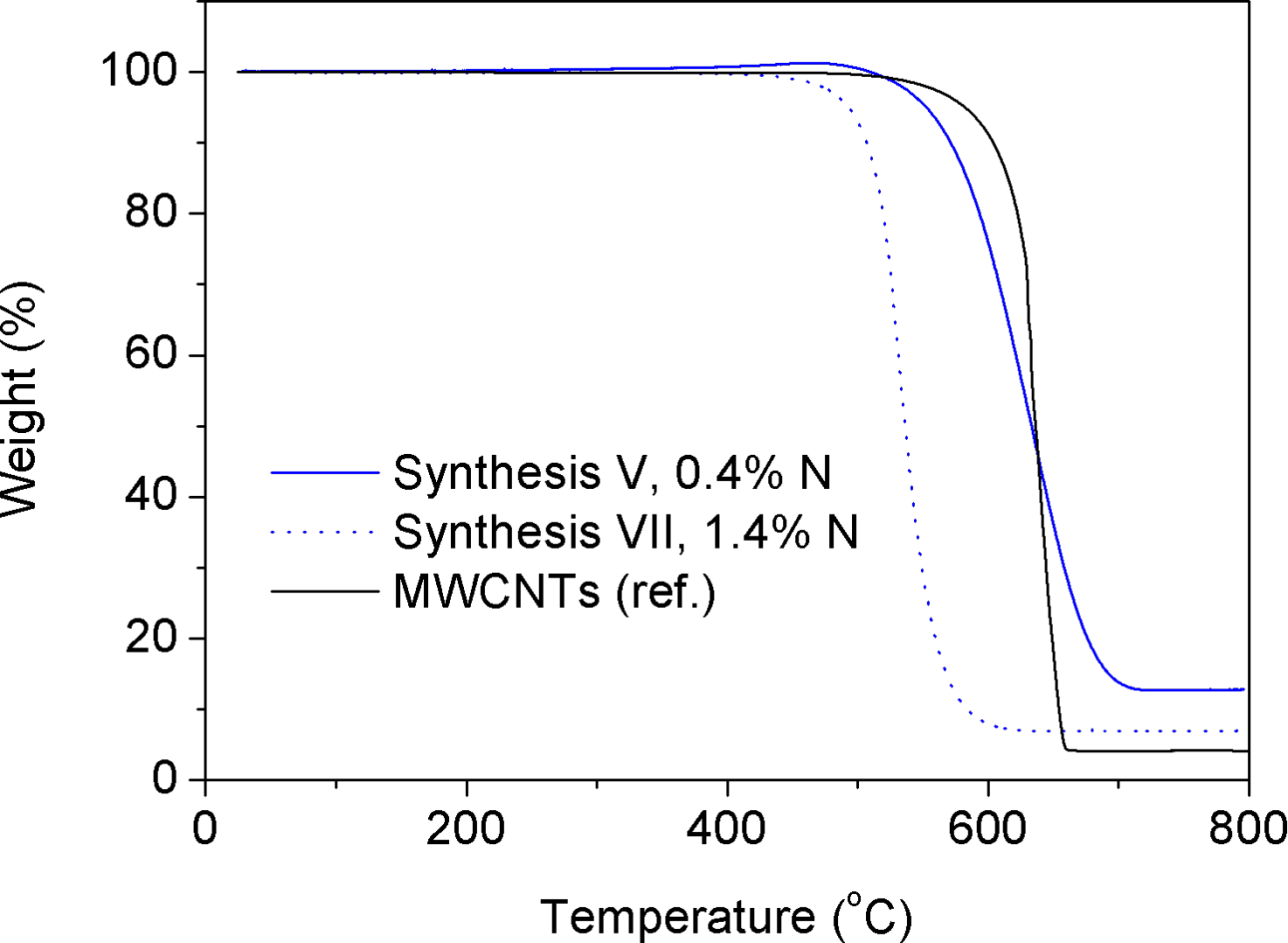
Overlaid TGA curves recorded in air for N-CNTs (*Synthesis V* and *VII*) and MWCNTs (*Ref. Synthesis*).

**Thermal stability.** Thermogravimetric analysis (TGA) showed that all N-CNTs (for the sake of clarity, only *Synthesis V* and *VII* are presented here) were less thermally stable in air than their undoped counterparts (Figure 8).

Referring back to the TEM images, a higher number of disordered graphitic layers (not fully graphitized) could explain this behavior. There are a few factors that are possibly responsible for a lower thermal stability of the N-CNTs as compared to pristine CNTs. The main point seems to be related to the defected morphology of the walls of the N-CNTs. A number of defects found in the N-CNTs by using Raman spectroscopy indicated that N-CNTs are less crystalline structures. Therefore, defect-inducing C–N bonds could generate a higher number of reactivity centres (‘hot-spots’) that are more susceptible to thermal cleavage and attack of oxygen. The higher number of ‘nanogrooves’, which translates into a higher surface area, could additionally enhance the accessibility of the outer nanotube walls. Moreover, the thermal resistivity in air was found to be inversely proportional to the nitrogen content in the N-CNTs. The maximum rate of combustion was found at 630 °C for both MWCNTs and N-CNTs (0.4% N), but for the latter oxidation initiated at ca. 20 °C earlier, and already at 530 °C for N-CNTs with 1.4% N. Furthermore, it must be emphasized that only single peaks were found in the DTA curves for N-CNTs whereas multiplets were recorded for MWCNTs indicating that the latter material was composed of more than one phase (see Figure S2, Supporting Information File 1). It was also noticeable that in the N-CNTs there was a fraction of iron more accessible to air, and therefore a slight increase in weight of the sample could be observed before the oxidation of the C-sp^2^ atoms has started. The residue left after completion of the analysis was composed of pure red Fe_2_O_3_, the weight of which enabled the determination of the Fe content in the nanotubes. These values were found to be in agreement with the elemental analysis discussed earlier.

**Figure 9:**
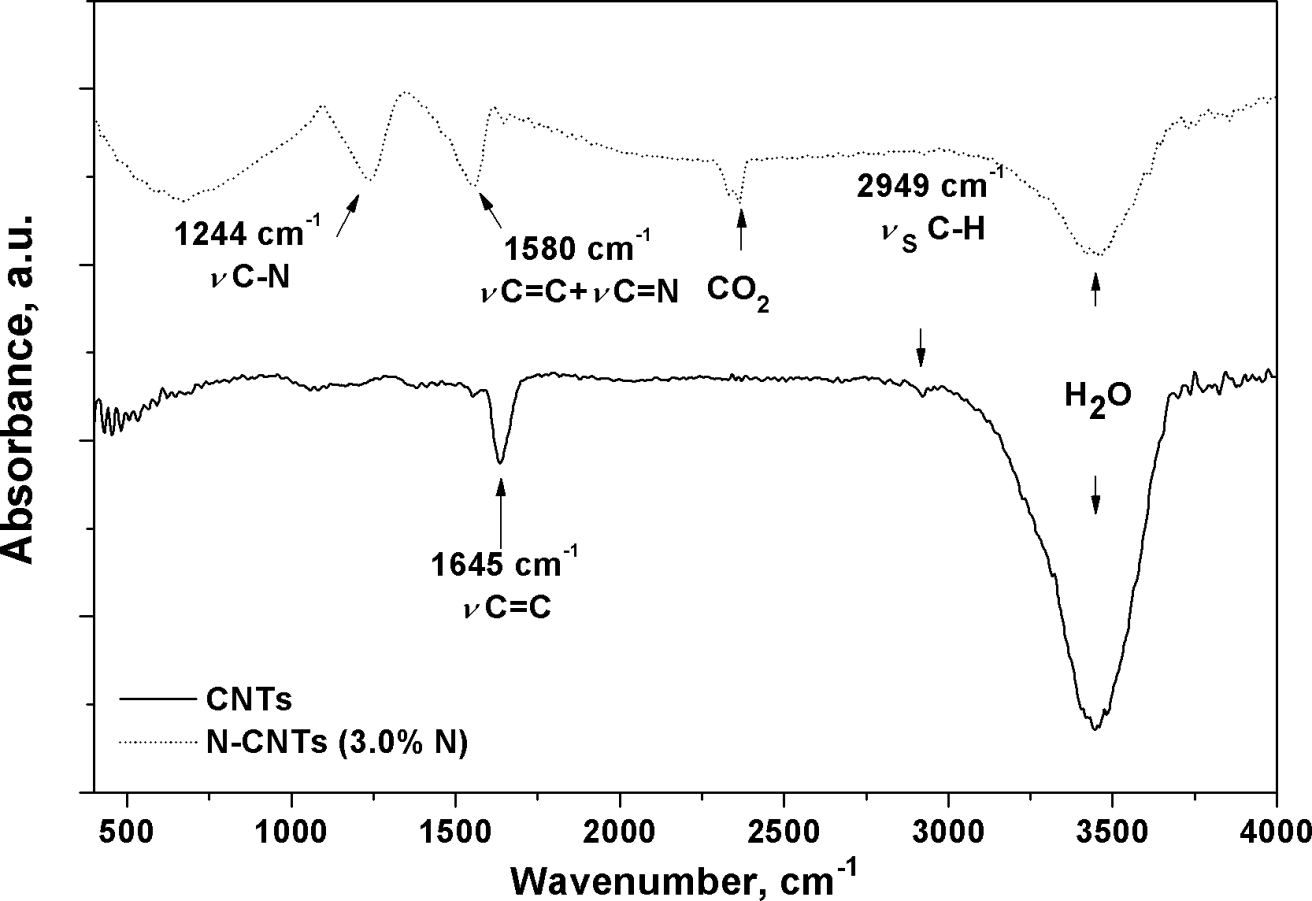
FT-IR spectra of CNTs and N-CNTs (*Synthesis VIII*).

**Spectral properties of N-CNTs.** FT-IR was applied to characterize the functional groups in the N-CNTs. Figure 9 shows the FT-IR spectra of MWCNTs and N-CNTs (*Synthesis VIII*) with main peaks at 1645, 2949 and 1244, 1580 and 2937 cm^−1^, respectively.

For MWCNTs the absorption band at 1645 cm^−1^ was assigned to the stretching of C=C bonds in the graphene layers (νC=C). The peak at 2949 cm^−1^ corresponds to the symmetric stretching of C–H bonds in carbonaceous material (ν_s_C–H). A similar signal for N-CNTs was absent, which indicated the lack of such impurities bound to the surface of the N-CNTs. In turn, the presence of C–N bonds could be identified by an intensive signal at 1244 cm^−1^, which possibly derived from the stretching of C–N bonds (νC–N). The incorporation of N-atoms into the graphitic lattice could be confirmed by a signal at 1580 cm^−1^ from a mixed stretching mode of C=N and C=C (νC=N + C=N), which was shifted to lower wavenumbers as compared to the signal from aromatic C=C bonds. These findings are in accordance with the previous reports [65–68], which demonstrated that the substitution of carbon atoms with nitrogen atoms in the sp^2^-networks induces a strong IR activity. Consequently, the absorption in the 1200–1600 cm^−1^ region would be expected if N-atoms were covalently bonded into the carbon network.

**Figure 10:**
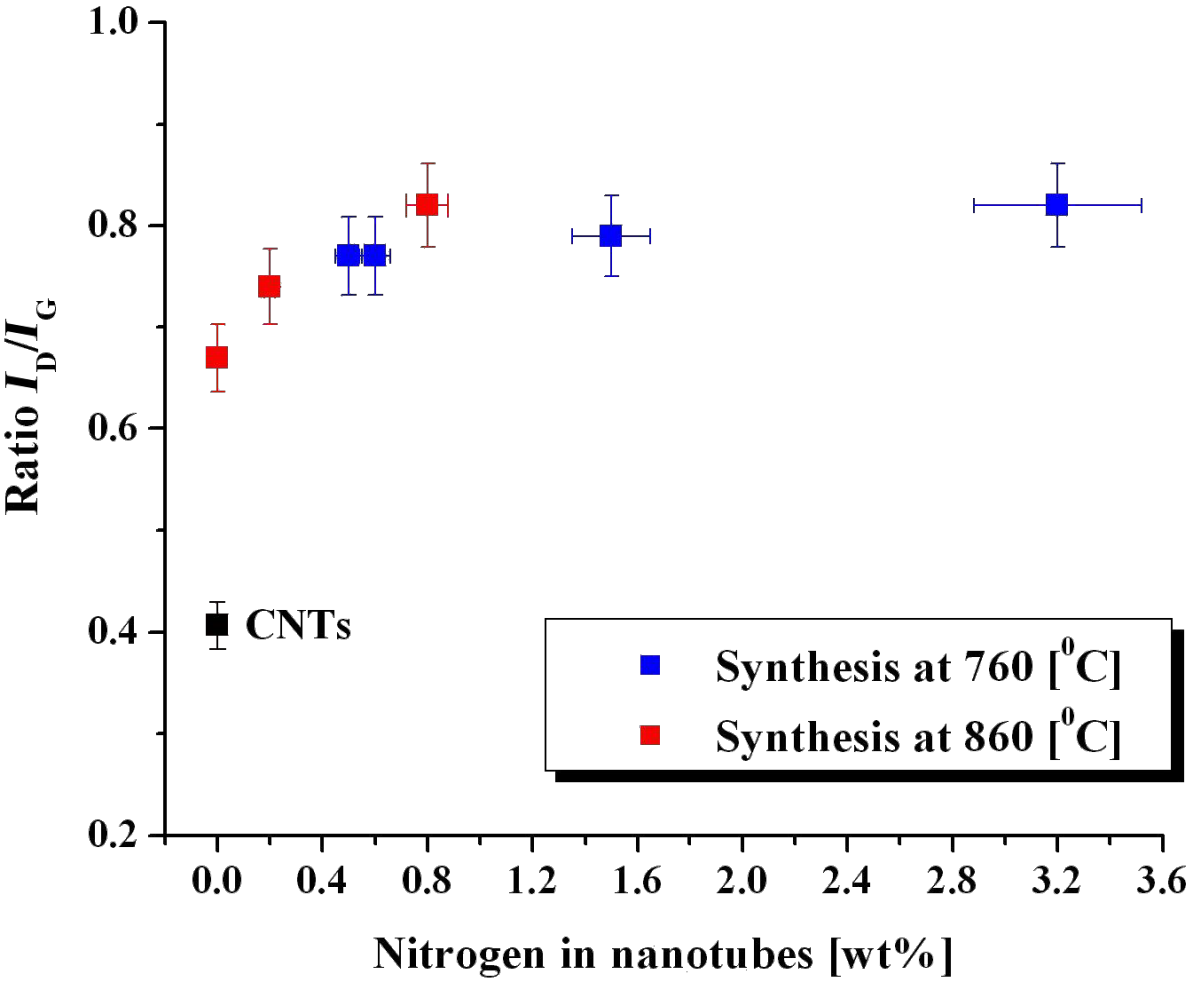
The ratio *I*_D_/*I*_G_ vs the level of N-doping at 760 and 860 °C, as compared to pristine MWCNTs.

Raman spectroscopy was used to determine a degree of crystallinity (graphitisation) of N-CNTs. It must be emphasized here that the ratio *I*_D_/*I*_G_ not only reflects of the presence of amorphous carbon (i.e., ‘cauliflowers’) but, particularly in our case, corresponds to the degree of graphitisation. In general, the ratio *I*_D_/*I*_G_ reflects a number of structural defects, the concentration of amorphous carbon and it is sensitive to doping. In the case of N-CNTs, the D-peak originates not only from structural defects but also from the covalent heteroatomic doping of the nanotubes. Firstly, changes in the positions of critical G- and D-peaks were found for N-CNTs (3% N) (ω_G_ = 1585 cm^−1^, ω_D_ = 1354 cm^−1^) as compared to MWCNTs (ω_G_ = 1576 cm^−1^, ω_D_ = 1351 cm^−1^). However, the critical changes appeared in the intensities of D-bands. The presence of nitrogen-based compounds during the growth of nanotubes affected their structure, which can be seen as an increase in the D-peak intensity. The relationship between the ratio *I*_D_/*I*_G_ and the nitrogen content in the N-CNTs is presented in Figure 10.

Importantly, in *Syntheses III* and *IV* also an increase in the *I*_D_/*I*_G_ ratio was found, although no nitrogen was detected by elemental analysis. This fact is of a great significance and it means that it is the presence of nitrogen species, which affects the growth of N-CNTs and, further, their morphology. Additionally, *I*_D_/*I*_G_ ratios increase with an increased N-doping at higher temperature. Continuing our insights into the mechanism of N-CNTs growth, we have investigated particular stages of the synthesis from the kinetic experiment. Raman spectra acquired from different stages of the N-CNTs synthesis confirmed that that N-CNTs preserve their chemical structure and morphology throughout the whole course of the synthesis since no significant changes, either in intensities or Raman shifts, were observed (see Table S1, Supporting Information File 1).

**Figure 11:**
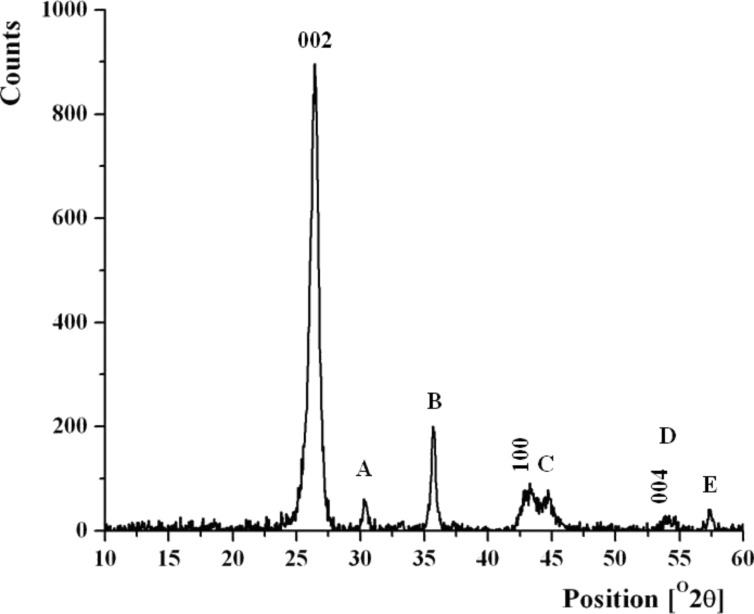
XRD pattern of N-CNTs (*Synthesis VIII*). The numbers are the hkl indices of the highest intensity peaks of graphite that coincide with the peaks from N-CNTs. The other peaks possibly refer to iron-based compounds.

**XRD analysis and growth mechanism.** Analysing the XRD patterns of N-CNTs (Figure 11), it was possible to identify peaks that correspond to the graphitic lattice. (002), (100) and (004) XRD reflections from pristine MWCNTs and N-CNTs match the values of graphite (see Table S1, Supporting Information File 1). Furthermore, there were other peaks observed for N-CNTs, which were absent in the XRD patterns acquired from pristine MWCNTs. By comparing 2θ values for those peaks with the reference XRD data [69], several iron based compounds could be assigned as phases accompanying the nanotubes. The most intensive reflections at 2θ = 42.9, 44.7 and 49.9° could be assigned to α-Fe (110) and γ-Fe (111, 200), respectively. The peak at 2θ = 35°, of the second highest intensity, matches several iron oxides, i.e., FeO, Fe_2_O_3_ and Fe_3_O_4_. The iron oxides in all of their possible crystallographic structures have nearly identical XRD patterns and match well with the peaks marked as A, B, C and D. Likewise, FeSiC, Fe_8_Si_2_C and FeSiO match with the peaks A and B. These compounds also have several peaks in the region of C, D and E. The XRD peaks of Fe_3_C overlap with the (002) reflection of graphite and also match with the B, C and E region. Most of the reflections were found to be relatively broad, hence they could originate from different phases. It is not possible to unequivocally identify those phases solely by analysing the XRD patterns.

**Figure 12:**
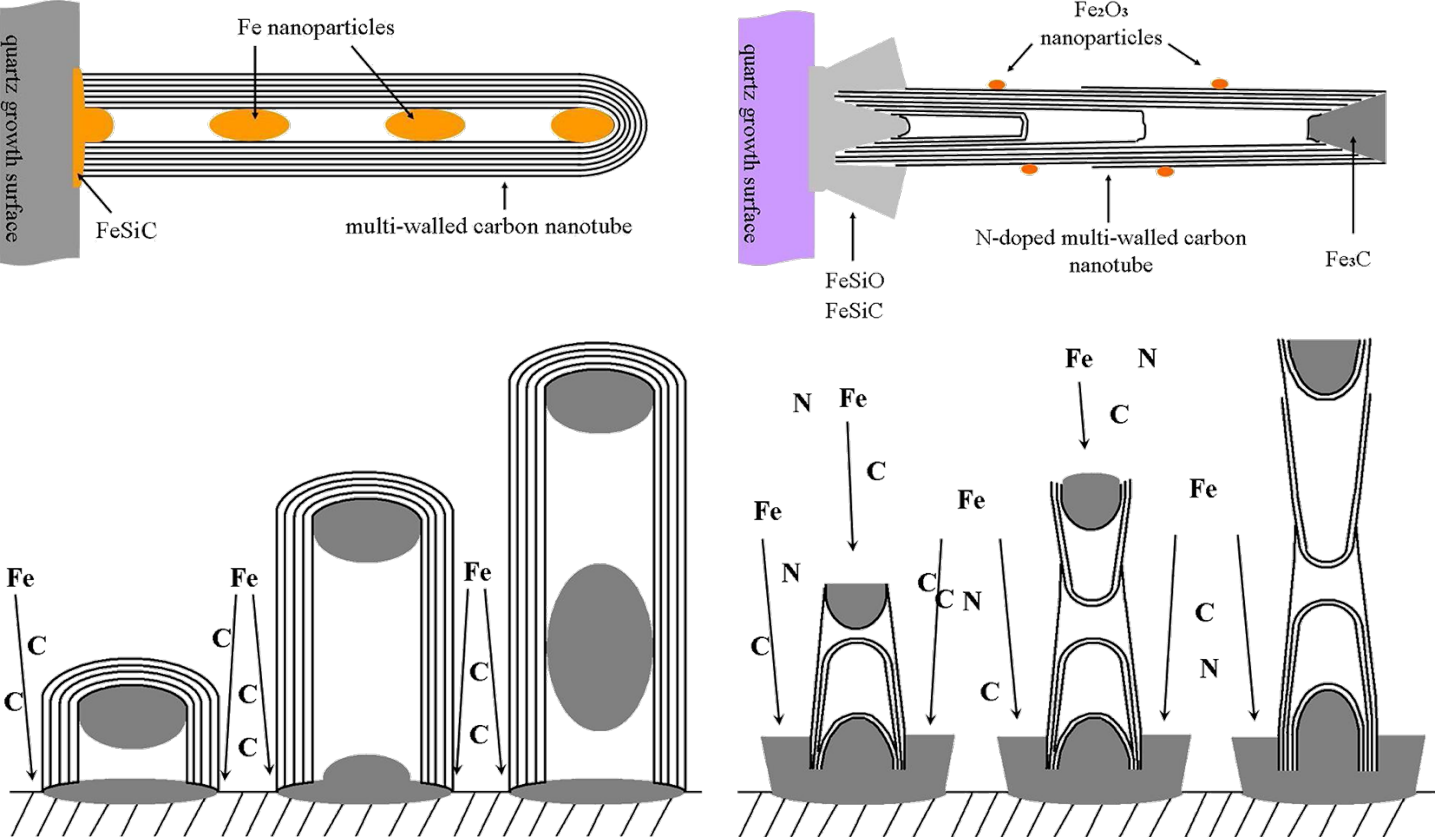
Models of MWCNT (left) and N-CNT (right) with metal particles differently distributed along the nanotube core (upper panel) and schematic presentation of a possible growth model for ‘base’ growth mechanism of MWCNTs and ‘mixed base and tip’ growth mechanism of N-CNTs (lower panel).

Based on the combination of TEM and XRD analysis, a model of MWCNT and N-CNT that contain nanoparticles of the catalyst was proposed (Figure 12, upper panel). For MWCNT, the metal particles were always encapsulated in the core of the nanotubes. It is most likely that the particles in the core of the nanotube are composed of pure iron and iron carbide. As XRD analysis revealed these particles were not oxidized because of the shielding from the carbon shell. The particle at the growth surface is an active catalyst particle (‘base’ growth mechanism) [70] and it is an iron silicon carbide phase. In case of N-CNTs, the catalyst particles found just underneath the nanotube surface are α-Fe (if encapsulated by few graphene layers) or Fe*_x_*O*_y_* (if the metal particle was not entirely protected by a graphene shell, and once exposed to air it was oxidized). The catalyst particle at the nanotube tip is probably Fe_3_C due to the catalytic growth of nanotubes and mainly carbon diffusion through the metal particle. The particle at the base (growth substrate) of the nanotube is likely to be iron silicon carbide and iron silicon oxide since it was in the contact with the silica quartz substrate.

In the nitrogen-assisted growth of nanotubes, the mechanism is more complicated than the ‘base’ growth of N-CNTs (Figure 12, lower panel). Since iron particles were observed at both the bottom and the top of individual nanotubes and no signal from iron was recorded from the middle part of the nanotubes, N-CNTs must grow in a mixed ‘base and tip growth’ mechanism, although primarily in a base-type growth [49]. We have already presented a direct evidence for the partial tip mechanism of the growth of N-CNTs [49]. We showed there that shape and position of the catalyst in the final N-CNT product can be explained with the following stages of the nanotube growth: (1) a formation of graphite nucleus and then a ‘carbon belt’, which fully encircles the catalyst nanoparticle, (2) as the ‘N-CNT belt’ grows, it stretches the catalyst particle (the catalyst is continuously replenished from the bottom) to form into the shape of a ‘sand clock’, (3) the catalyst particle divides and the growth continues from both sides, (4) the new layers are then formed internally and these break off, cap-wise, from the growing and receding catalyst particles to yield the interconnected webs in core of the nanotube, and (e) the final shapes of catalyst residues at the tip and at the bottom of the nanotube are witnesses and remains of the growth process. It must be also emphasized here that catalyst particles were absent in the MWCNT tips.

**Figure 13:**
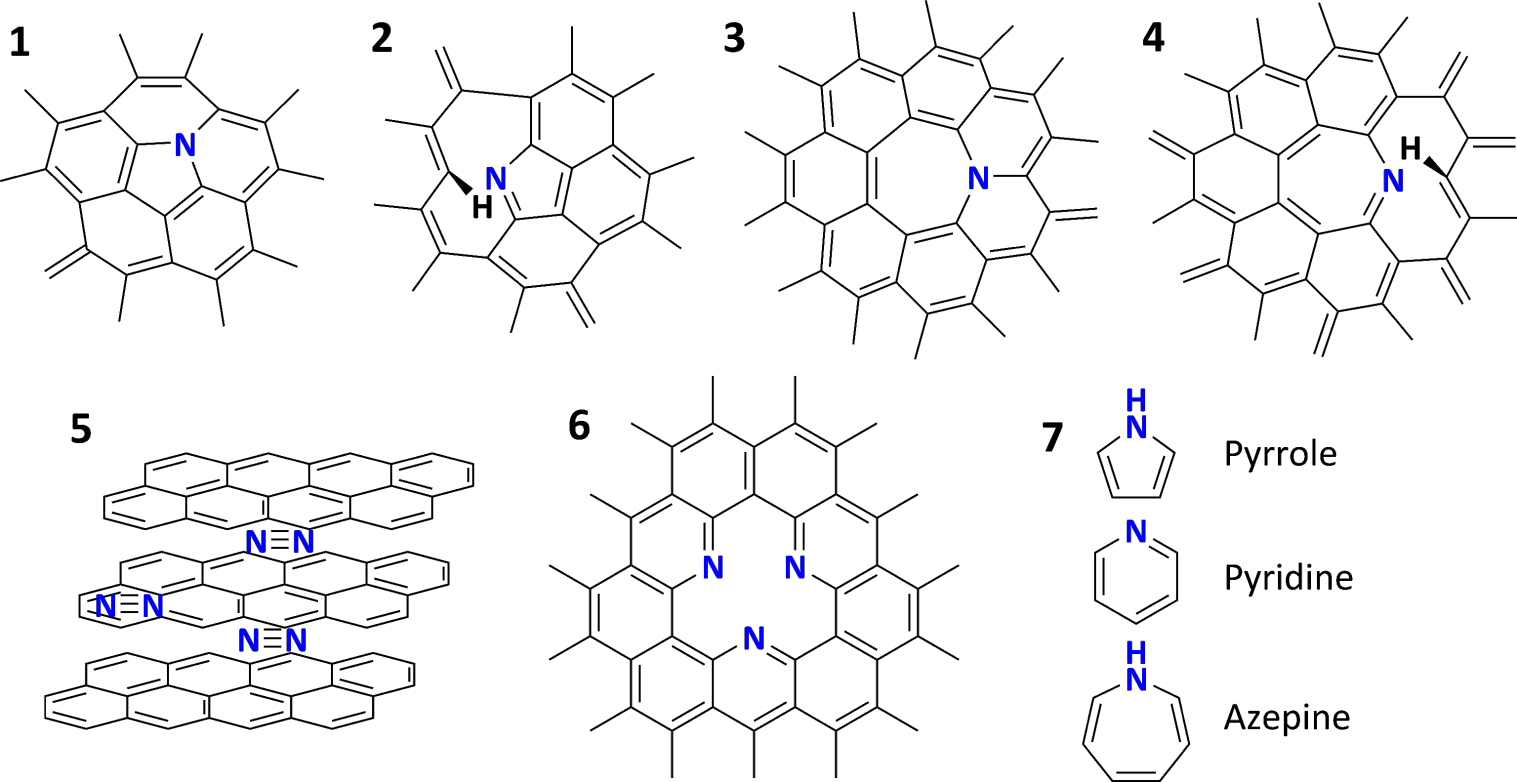
Examples of how nitrogen atoms can be incorporated into the graphene layer: (1) and (3) deformational substitutions; (2) and (4) deformational substitutions with a vacancy; (6) “nitrogen gap” (three ‘pyridinic’-like nitrogen atoms, that all lead to a higher degree of freedom of the lattice); (5) molecules of nitrogen entrapped between adjacent graphene layers. The types of nitrogen-doping can be referred to ‘pyridinic-‘, ‘pyrrolic-‘ and ‘azepinic-like’ in analogy to the nomenclature of simple heterocyclic molecules.

Based on results from FT-IR and Raman spectroscopy, and TG measurements, some important premises on the molecular structure of N-CNTs can be suggested. As it was shown, the interstitial nitrogen in graphite lattice leads to the development of defects. Even a difference of 0.5% between the C–C and C–N bond lengths in nanotubes (0.1422 nm and 0.1429 nm, respectively) leads to deformations and bending of the sp^2^-layers [30]. Partial deformations of the graphene walls were found to be caused by the formation of 5- and 7-membered rings in MWCNTs. This effect will be even more distinct for N-doped CNTs since 5- and 7-membered rings that contain nitrogen are thermodynamically more stable their C-homoatomic analogues [71]. The most probable ways of N-incorporation into graphene walls, including substitutions and substitutions with a simultaneous formation of vacancies structures, are presented in Figure 13. The presence of nitrogen or 5- and 7-member-ring defects is crucial for the formation of the cone structure of the N-CNTs. The vacancies also provide geometrical degrees of freedom to the structure of N-CNTs, which allows for the fulfilment of the crystallographic matching criteria (–ABAB–) between the layers. This is geometrically impossible in the case of MWCNTs.

## Conclusion

N-CNTs were synthesized via an injection c-CVD method. With the intension to grow the most aligned and dense arrays of N-CNTs, we have studied the parameters of the N-CNTs growth to find the concentration of pyrazine in the toluene/ferrocene solution and temperature as the critical parameters. The most dense and aligned N-CNTs were obtained when [Pz] of 30 and 45 wt % were used in a mixture with toluene and ferrocene. The nanotubes, grown via a ‘mixed base-and-tip’ growth mechanism at 760 °C, exhibited ‘bamboo-‘ or ‘membrane’-like (compartmental) morphology and a narrow diameter distribution of 43 ± 22 and 49 ± 19 nm. The highest areal density of up to 5 × 10^8^ N-CNTs per mm^2^ in the array was gained for nanotubes of OD equal to 26 ± 15 nm grown at [Pz] = 5 wt % and [FeCp_2_] = 9.6 wt %. These results make N-CNTs the perfect construction material for separation membranes of tuneable permeability. N-CNTs of a high N-doping level can be used as high surface area electrodes. Apart from this, the ‘bamboo’-like N-CNTs obtained here could provide a new opening in drug delivery systems due to their rigid ‘needle-like’ morphology. These N-CNTs with a high content of ferromagnetic nanoparticles could potentially serve as magnetically steerable drug carriers for the enhanced penetration of target cells in anticancer therapies [72–73].

## Experimental

**Synthesis.** The synthesis setup was composed of a pre-heater, a furnace, a quartz reaction tube, injection pump with a syringe, an inert gas flow-meter and an exhausts purifier. The pre-heater was assembled with a T-junction shape quarter inch quartz tube (Cambridge Glassblowing Ltd.) wrapped by a heating tape (electrothermal HT9, Fisher Scientific) made of resistive wires covered in a glass fibre fabric. The heating tape was operated with a digital controller (electrothermal, MC810). The temperature of the pre-heater was maintained at 180 °C and in order to avoid heat loss, it was insulated by a ceramic fibre cloth. The function of the pre-heater was to evaporate the carbon feedstock injected by a 50-mL gas-tight Hamilton syringe, which then was taken by pre-dried argon as the carrier gas (Air products, 99.995 %), into the reaction tube. The syringe was operated by the injection pump (Linton instrumentation, KD Scientific). A fused quartz reaction tube (silica, 99.99 %) of following dimensions: length 2000 mm, OD 17 mm, ID 14 mm was introduced into the furnace (Lenton Thermal Designs CSC12, split 3-zone tube furnace). The exhausts from the reaction tube were directed through a bottle containing activated carbon and another one with silicon oil.

**Characterisation.** The elemental analyses of dried samples (atmospheric pressure desiccator containing silica gel) were performed by using an Exeter Analytical CE-440 CHN Elemental Analyzer. A JEOL 6340F FEG SEM was used with an accelerating voltage of 5 kV. A secondary electron imaging (SEI) detector was used in all cases. TEM imaging of nanotube samples was performed on JEOL 200CX (tungsten filament, operated at 200 kV), JEOL 2000FX (LaB6 electron source, operated at 200 kV), FEI Tecnai F20-G2 (field emission gun, operated at 200 kV) and JEOL 4000EX-II (LaB_6_ electron source, operated at 400 kV) transmission electron microscopes depending on characterization requirements. Prior to imaging the samples were dispersed in diethyl ether in an ultrasonication bath and dropped onto 400 mesh copper grids covered with a holey carbon film. The nanotube samples were investigated by using a RM1000 (Ramascope-1000 system) Raman microscope with a spectral resolution of 0.1 cm^−1^ and a spatial resolution of the X/Y/Z stage of 1 µm. An argon ion laser (green, λ = 514.5 nm) was used in all cases. FT-IR analyses were performed by using a Perkin Elmer spectrometer in the range from 0.44 eV (2778 nm, 3600 cm^−1^) to 0.1 eV (12500 nm, 800 cm^−1^) from KBr pellets. TGA analyses were carried out in air at a heating rate of 10 °C·min^−1^ by using a TGA Q500. XRD studies were performed on a Philips GEN4 diffractometer at 40 kV and 40 mA, which covered the 2θ angles from 8 to 60°. Cu Kα radiation (λ = 0.154 nm) was used. The nanotube powder sample was supported by a single crystal silicon holder.

## Supporting Information

File 1 Additional experimental data.
