# Supplementary material for: En route to controlled catalytic CVD synthesis of densely packed and vertically aligned nitrogen-doped carbon nanotube arrays
Source: Beilstein J Nanotechnol. 2014 Mar 3;5:219–33. doi: 10.3762/bjnano.5.24 (PMC3944053; doi:10.3762/bjnano.5.24)
Supplement: File 1 — Additional experimental data. [file Beilstein_J_Nanotechnol-05-219-s001.pdf]

Supporting Information

for

En route to controlled catalytic CVD synthesis of densely packed and vertically aligned nitrogen-doped carbon nanotube arrays

Slawomir Boncel[*1], Sebastian W. Pattinson[2], Valérie Geiser[2], Milo S.P. Shaffer[3] and Krzysztof K.K. Koziol[*2]

Address: [1]Department of Organic Chemistry, Biochemistry and Biotechnology, Silesian University of Technology, Krzywoustego 4, 44-100 Gliwice, Poland, [2]Department of Materials Science and Metallurgy, University of Cambridge, 27 Charles Babbage Road, Cambridge CB3 0FS, United Kingdom and [3]Imperial College London, Department of Chemistry, London SW7 2AZ, United Kingdom

Email: Slawomir Boncel* - slawomir.boncel@polsl.pl;

Krzysztof K.K. Koziol* - kk292@cam.ac.uk

* Corresponding author

Additional experimental data

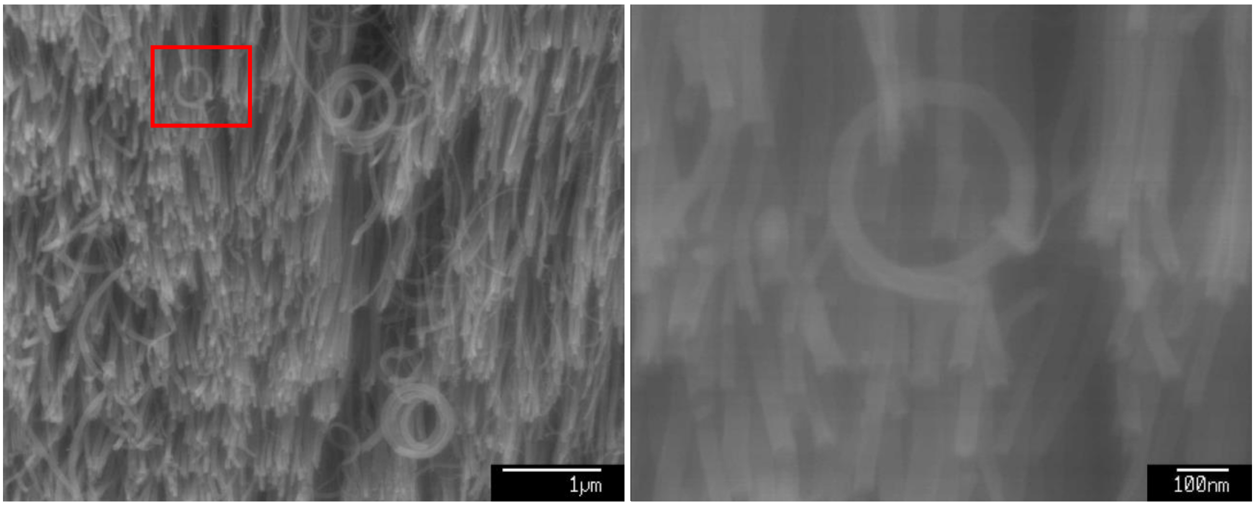

Figure S1: Spiral and some entangled N-CNTs (left) from *Synthesis V*, and magnification of indicated area.

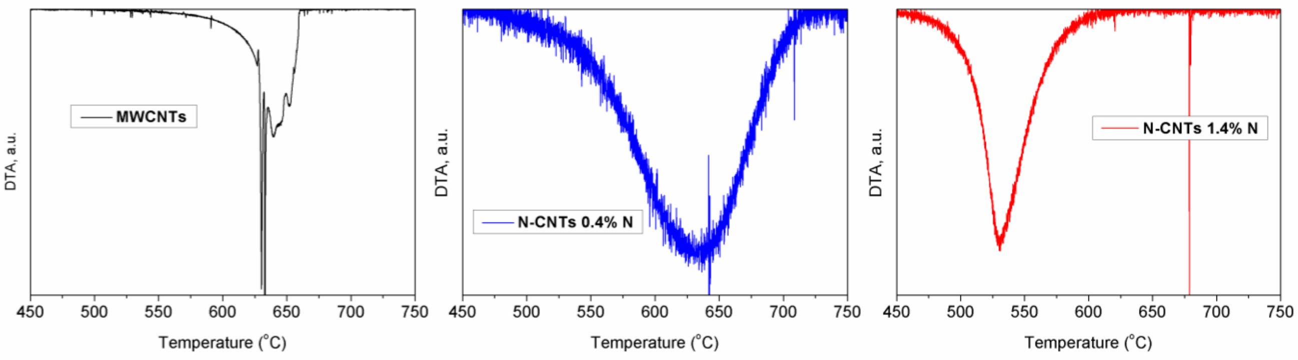

Figure S2: DTA curves for MWCNTs, N-CNTs (0.4% N) and N-CNTs (1.4% N). For MWCNTs of a high degree of inhomogeneity – various fractions (ca. five) of carbon and carbonaceous material undergoing combustion with a maximum rate at different temperatures; Contrarily, N-CNTs show a uniform behaviour when heated in air (10 °C·min^{-1}) but are more susceptible to thermal degradation because of a high number of distortions/deformations of the graphene walls.

Table S1: Raman shifts of N-CNTs as a function of the time of synthesis as compared to MWCNTs; no significant changes either in position of the critical peaks or changes in the ratio I_D/I_G could be found throughout the course of synthesis.

	G'-Band			G-Band			D-Band			I_D/I_G
	$\omega_{G'}$	$I_{G'}$	$\Delta\omega_{G'}$	ω_G	I_G	$\Delta\omega_G$	ω_D	I_D	$\Delta\omega_D$	
N-CNTs_1h	2645.8	780.2	7.4	1573.6	1393.9	−1.5	1333.9	1211.5	7.5	0.87
N-CNTs_2h	2647.0	508.4	8.6	1572.1	925.9	−2.9	1333.9	841.2	7.5	0.91
N-CNTs_3h	2671.6	559.0	33.2	1582.3	1171.0	7.3	1327.9	1162.6	1.5	0.99
N-CNTs_4h	2660.5	629.2	22.2	1575.0	1232.8	0.0	1329.4	1160.1	3.0	0.94
MWCNTs	2638.4	3632.0	0.0	1575.0	3075.7	0.0	1326.4	1313.9	0.0	0.43

Table S2: List of graphite-derived XRD reflections acquired from N-CNTs vs MWCNTs.

MWCNTs			N-CNTs		
Normalised intensity	Bragg angle (°2θ)	*hkl*	Normalised intensity	Bragg angle (°2θ)	*hkl*
100	25.8	002	100	26.3	002
2	42.3	100	9	42.2	100
2	53.7	004	3	54.6	004
